# Bioavailability of fatty acids from krill oil, krill meal and fish oil in healthy subjects–a randomized, single-dose, cross-over trial

**DOI:** 10.1186/s12944-015-0015-4

**Published:** 2015-03-15

**Authors:** Anton Köhler, Essi Sarkkinen, Niina Tapola, Tarja Niskanen, Inge Bruheim

**Affiliations:** Preventive Cardiology, Medizinische Klinik I, University of Munich, Ziemssenstr. 1, Munich, D-80336 Germany; Oy Foodfiles Ltd, Kuopio, Finland; University of Eastern Finland, Kuopio, Finland; Olympic Seafood, Fosnavaag, Norway

**Keywords:** Omega-3 fatty acids, Eicosapentaenoic acid, Docosahexaenoic acid, Krill oil, Krill meal

## Abstract

**Background:**

Krill contains two marine omega-3 polyunsaturated fatty acids, eicosapentaenoic acid (EPA) and docosahexaenoic acid (DHA), mainly bound in phospholipids. Typical products from krill are krill oil and krill meal. Fish oils contain EPA and DHA predominantly bound in triglycerides. The difference in the chemical binding of EPA and DHA has been suggested to affect their bioavailability, but little is known on bioavailability of EPA and DHA in krill meal.

This study was undertaken to compare the acute bioavailability of two krill products, krill oil and krill meal, with fish oil in healthy subjects.

**Methods:**

A randomized, single-dose, single-blind, cross-over, active-reference trial was conducted in 15 subjects, who ingested krill oil, krill meal and fish oil, each containing approx. 1 700 mg EPA and DHA. Fatty acid compositions of plasma triglycerides and phospholipids were measured repeatedly for 72 hours. The primary efficacy analysis was based on the 72 hour incremental area under the curve (iAUC) of EPA and DHA in plasma phospholipid fatty acids.

**Results:**

A larger iAUC for EPA and DHA in plasma phospholipid fatty acids was detected after krill oil (mean 89.08 ± 33.36% × h) than after krill meal (mean 44.97 ± 18.07% x h, p < 0.001) or after fish oil (mean 59.15 ± 22.22% × h, p=0.003). Mean iAUC’s after krill meal and after fish oil were not different. A large inter-individual variability in response was observed.

**Conclusion:**

EPA and DHA in krill oil had a higher 72-hour bioavailability than in krill meal or fish oil. Our finding that bioavailabilities of EPA and DHA in krill meal and fish oil were not different argues against the interpretation that phospholipids are better absorbed than triglycerides. Longer-term studies using a parameter reflecting tissue fatty acid composition, like erythrocyte EPA plus DHA are needed.

**Trial registration:**

NCT02089165

## Background

In Western countries, like Germany or the United States, mean levels of the marine omega-3 fatty acids eicosapentaenoic acid (EPA) and docosahexaenoic acid are below the range defined as optimal [1]. In large parts, this is due to a decline in uptake of EPA and DHA [1], but also due to the fact that, under Western dietary conditions, only a very low percentage (if any) of alpha-linolenic acid can be converted to EPA, and almost no conversion to DHA occurs [2,3]. Therefore, sources of EPA and DHA have to be ingested directly. Since the production of fish oil cannot be increased easily, other sources of EPA and DHA are currently investigated.

One of them is krill, a shrimp-like crustacean that feeds off algae in deep ocean waters. Krill is rich in both EPA and DHA. In Krill, 30–65% of EPA and DHA are in phospholipid fatty acids, while in fish or fish oil, EPA and DHA are mainly in triglycerides (sn-2 position) [4,5]. In contrast to triglycerides, phospholipids are amphiphilic and thus have emulsifying features, which might contribute to enhanced absorption. However, as discussed in more detail elsewhere, bioavailability of EPA and DHA depends on a host of factors, like composition of food ingested with EPA and DHA (if any), other matrix effects, and others [5,6].

Thus, the bioavailability of any new source of EPA and DHA has to be investigated in humans. Indeed, some reports have demonstrated that EPA and DHA in krill oil are bioavailable, and some indicating superior bioavailability of EPA and DHA in krill oil, as compared to EPA and DHA in fish oil [4,5,7]. However, to the best of our knowledge (medline search on Dec 17, 2014), bioavailability of EPA and DHA in krill meal remained to be investigated in humans.

Therefore, we compared the acute bioavailability of EPA and DHA in two different krill products, krill meal (Rimfrost Genuine, 40% oil content) and krill oil (Rimfrost Sublime), to the bioavailability of EPA and DHA in fish oil. In a randomized, cross-over trial, we provided a single dose of about 1,700 mg EPA and DHA to human volunteers.

## Subjects and methods

### Study subjects

Study participants were recruited through an advertisement in the local newspaper from the area of the Northern Savo (Kuopio, Finland). Inclusion criteria were: (1) healthy female and male subjects, (2) age between 18 and 65 years, (3) body mass index (BMI) between 18.5 and 30 kg/m^2^. Exclusion criteria were: (1) use of any medication with a potential to affect the bioavailability of fatty acids (e.g. lipid-lowering medication); (2) familial hypercholesterolemia, marked combined hyperlipidemia and any other condition that might impair fat absorption (e.g. chronic pancreatitis, pancreatic lipase deficiency syndrome); (3) type 1 or type 2 diabetes; (4) cancer or any other malignant disease within the past five years; (5) use of cyclic or any other discontinuous hormone replacement therapy; (6) ≥ once a week of fatty fish (e.g. salmon, herring, sardines, mackerel, vendace, anchovy) consumption; (7) regular consumption of omega-3 supplements within 4 weeks prior the randomization; (8) smoking; (9) alcohol consumption of more than 15 units per week (1 unit=33 cl beer/12 cl wine/4 cl sprits providing about 12 g alcohol); (10) females who are pregnant, breast-feeding or intend to become pregnant; (11) hypersensitivity to any of the components of the test product (including fish allergy); and (12) other medical reasons judged by the investigator to render the subject unsuitable for the study participation.

The trial was approved by the Research Ethics Committee of the Hospital District of Northern Savo (Kuopio, Finland), registered on Clinicaltrials.gov (NCT02089165), and conducted according to the Declaration of Helsinki and Good Clinical Practice. Written informed consent was obtained from all subjects before any study related procedures.

### Study design

A randomized, single-dose, single-blind, cross-over, active-reference intervention trial was performed in healthy subjects, comparing Antarctic Krill Oil, Antarctic Krill Meal, and Fish Oil. The primary endpoint was the proportion of EPA + DHA in plasma phospholipid fatty acids during 72 hour follow-up after ingestion, measured as incremental area under the curve (iAUC_PL_). Secondary endpoints were the incremental area under the curve of EPA + DHA in plasma triglyceride fatty acids (iAUC_TG_); maximum increases of EPA, DHA and EPA + DHA from baseline and time to maximum EPA, DHA and EPA + DHA in plasma phospholipid fatty acids and triglyceride fatty acids, each during 72 hours after ingestion.

Permuted block randomization was performed using the incomplete 3 × 3 Latin square design (Table 1). Each subject was assigned to one of three sequences of interventions in a non-concealed way (Table 1). Altogether 17 healthy subjects were randomized, and 15 subjects participated all three 72-hour tests periods as randomized. Each participant received each treatment 13–29 days apart.

**Table 1:** **Latin square design**

Sequence 1	Krill oil supplement (A)	Fish oil supplement (B)	Krill meal (C)
Sequence 2	Fish oil supplement (B)	Krill meal (C)	Krill oil supplement (A)
Sequence 3	Krill meal (C)	Krill oil supplement (A)	Fish oil supplement (B)

Blood samples were taken after a 10–12 hour overnight fast 20 min before breakfast and at 2, 4, 6, 8, 12, 24–25, 48–50 and 72–74 hours after start of breakfast ingestion. Subjects were served lunch, snack, dinner and supper immediately after blood sampling at 4, 6, 8 and 12 hour after start of breakfast ingestion, respectively. Subjects spent the test days at the study center until supper was served, and consumed. After supper, subjects went home and returned to the study center the following three mornings for blood sampling. The participants were not permitted to consume any food or drink (except for one glass of water in the morning before sampling) after supper until the blood sample was taken at 24–25 hour after start of the breakfast ingestion.

#### Standardized evening meal preceeding the test days

Participants were provided a standardized evening meal the day before the test, consisting of 300 g commercial ham and potato casserole (Saarioinen Oy, Kangasala, Finland), tomato/cucumber and 300 ml of tap water. The casserole was provided by the study center and the participants were advised to warm up the casserole before eating and measure the water dose. In addition, the participants were permitted to consume optional amount of tomato and or cucumber with the evening meal but were advised to consume the same amount at each time. The participants were advised to eat the evening meal just before the start of the overnight fast.

#### Standardized breakfast and products tested

The standardized breakfast meal included 2 open sandwiches (rye bread, fat spread, cheese, cucumber, tomato), one boiled egg, 150 g of yoghurt and 400 ml of tap water. The standardized breakfast (without the study product) contained 2232 kJ, 43 g carbohydrates, 29 g fat and 22 g protein. In addition, participants consumed one of the following three: 1) 16 capsules of antarctic krill oil (Rimfrost Sublime™, Olympic Seafood AS, Fosnavåg, Norway), 2) 12.3 g of antarctic kill meal (Rimfrost Genuine™, Olympic Seafood AS, Fosnavåg, Norway), 3) 10 capsules of fish oil supplement (Bioteekin Kalaöljykapseli, Suomen Bioteekki Oy, Raisio, Finland). The 12.3 g dose of krill meal was mixed with 150 g of yoghurt (Valiojogurtti® Sileä vadelma HYLA®, Valio Oy, Oulu, Finland) before serving. Breakfast was consumed under supervision of the study nutritionist. The mean duration of breakfast consumption was 15 ± 5 min, when krill oil supplement was consumed, 15 ± 8 min, when krill meal was consumed and 13 ± 3 min when fish oil supplement was consumed.

The test products were stored in dark, at 4–8°C at the study center. The portion size of both test products and active reference each provided about 1 700 mg of omega-3 polyunsaturated fatty acids (Table 2). The fatty acid profiling of the krill products and fish oil supplement was done by Nofima BioLab (Fyllingsdalen, Norway). The fish oil supplement used in this study was in triglyceride form and was made from sardines and mackerels.

**Table 2:** **omega-3 polyunsaturated fatty acid, EPA and DHA content of the study products**

**Test product**	**Dose**	**Total fat**	**Total omega-3 PUFA**	**EPA**	**DHA**	**EPA + DHA**
Krill oil	16 capsules	8000 mg	1704 mg	896 mg	504 mg	1 400 mg
Krill meal	12.3 g	5535 mg	1710 mg	692 mg	410 mg	1 102 mg
Fish oil	10 capsules	5000 mg	1730 mg	875 mg	580 mg	1 455 mg

#### Standardized lunch, snack, dinner and supper during the test days

Subjects were served ad libitum standardized lunch, dinner and supper during the first day after blood sampling at 4, 8 and 12 hours after start of breakfast ingestion, respectively. For each study subject the similar pre-weighed portions of foods were served and any leftovers were weighed with a digital scale (Kern PCB, Kern&Sohn GmbH, Balingen-Frommern, Germany). The amounts of foods consumed at each meal were replicated and provided for the subjects at day two and three. The same snack was served after blood sampling at 6 hours after the start of the breakfast ingestion at each test day.

The lunch meal consisted of commercial vegetable soup, rye and oat bread, fat spread, cheese, cucumber and tomato slices. The snack consisted of one pear (or orange, if the subject was allergic to pear) and 200 ml of coffee and optional milk and sugar. Dinner consisted of commercial chicken pasta casserole, rye and oat bread, fat spread, cheese, cucumber and tomato slices. Supper consisted of rye and oat bread, fat spread, cheese, ham, cucumber and tomato slices.

#### Liquid intake

Only tap water and 200 ml coffee were provided to the subjects during the first 24 hours of the test. Participants consumed standardized amounts of tap water with breakfast (400 ml) and supper (250 ml). In addition, participants were asked to consume amounts of tap water identical to the first day in the two subsequent days.

#### Background diet, concomitant treatment and medication

At the beginning of the run-in period, participants were advised to maintain their lifestyles (physical activity, alcohol and dietary supplement consumption) and dietary habits (especially consumption of vegetable oils) throughout the study. However, consumption of fatty fish (e.g. salmon, herring, sardines, mackerel, vendace, anchovy) and/or omega-3 fatty acid supplements was forbidden during all study periods. The subjects were instructed to avoid vigorous physical activity, alcohol consumption, unusual foods and heavy meals during 24 hour prior to each test.

### Laboratory methods

Blood samples were centrifuged at 2000 × g for 10 minutes at 10°C after collection. Plasma was immediately frozen at -70°C for fatty acid analysis. The samples were shipped frozen under dry ice to the laboratory (Omegametrix GmbH, Martinsried, Germany) after completion of the study.

Lipids were extracted and triglycerides and phospholipids were purified by use of Sep−Pak C-18 mini cartridges (Waters, Eschborn, Germany). Fatty acid methyl esters were formed by acid hydrolysis, and were analyzed by capillary gas–liquid chromatography on a GS2010 Gas Chromatograph (Shimadzu, Duisburg, Germany) equipped with a SP2560, 100-m column (Supelco, Bellefonte, Pennsylvania, USA) using hydrogen as carrier gas. Fatty acids were identified by comparison with a standard mixture of fatty acids. The results are given as percentage of total identified fatty acids.

Blood samples for hematology and clinical chemistry were taken at the screening visit and at the end of each test (at 72–74 h after start of breakfast ingestion) after a 10–12 h overnight fast. Blood count, serum creatinine and gamma-glutamyl transferase concentrations were assessed and analyzed at United Medix Laboratories Ltd (Espoo, Finland) with standardized methods. At the screening visit, serum thyroid-stimulating hormone level was also analyzed to ensure the health status of the participants.

### Statistical analysis

The planned sample size was based on the primary endpoint, the proportion of EPA + DHA in plasma phospholipid fatty acids during 72 hour follow-up after ingestion, measured as incremental area under the curve (iAUC_PL_). The standard deviation of the iAUC of phospholipid EPA + DHA proportion was assumed to be 34.7% × h [8]. A sample size of 15 subjects was planned in order to be able to detect a difference of 25% × h in mean iAUC of phospholipid EPA + DHA proportion with a probability of 80% at α level of 0.05.

The intention to treat (ITT) population was defined as all randomized subjects who received at least one dose of study product.

Incremental areas under the curves of EPA + DHA in plasma phospholipids and plasma triglycerides were calculated for each person during each intervention period according to the trapezoidal rule using the RStudio (Version 0.98.1028–© 2009–2013 RStudio, Inc.), ignoring the area beneath the fasting percentage.

Maximum increase from baseline (0 min) in percentage of EPA, DHA and EPA + DHA in plasma triglycerides and phospholipids were calculated for each person during each intervention period by deducting the individual baseline (0 min) proportion from the individual observed peak proportion (cmax) during the 72-h follow-up after administration of the study product.

The times (tmax) from baseline to maxima of EPA, DHA and EPA + DHA in plasma triglycerides and phospholipids were identified for each subject at each intervention period.

Results are presented as means and standard deviations. Statistical differences were calculated using paired *t* test for the comparison of baseline levels and calculations mentioned above. Differences with *p* values < 0.05 were considered statistically significant. Because of multiple testing, Bonferroni adjustments were used and *p* values < 0.017 were considered statistically significant for comparison of the results after intervention with the three different study products. Data were examined by IBM SPSS Statistics for windows (release 18.0 Chicago, IL, USA).

## Results

### Screening

**Figure 1:**
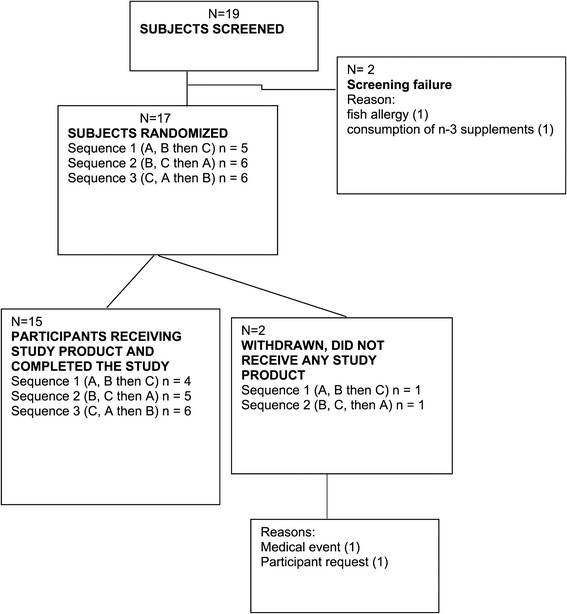
**Study flow diagram.**

A total of 19 subjects signed informed consent, and was screened; 17 subjects were randomized. Characteristics of participants are presented in Table 3. Altogether two subjects dropped out of the study during the run-in period. One due to a personal reason and the other participant for medical reasons (was prescribed ear surgery). A total of 15 participants started the first intervention and all 15 completed the study (Figure 1).

**Table 3:** **Baseline lifestyle characteristics of the participants (n=15)**

		**Subjects**
**Vitamin and or mineral supplement**	yes	11 (73%)
	no	4 (27%)
**Other food supplement**	yes	6 (40%)
	no	9 (60%)
**Alcohol consumption**	daily	0 (0%)
	weekly	7 (47%)
	monthly	5 (33%)
	rarely/ not at all	3 (20%)
**Commuting (time spent walking or cycling)**	out of work or working at home	14 (93%)
	go by motor vehicle	0 (0%)
	<15 min a day	0 (0%)
	15–30 min a day	0 (0%)
	30–60 min a day	1 (7%)
	>60 min a day	0 (0%)
**Exercise**	Daily	2 (13%)
	4-6 times a week	6 (40%)
	3 times a week	2 (13%)
	2 times a week	2 (13%)
	once a week	1 (7%)
	2-3 times a month	1 (7%)
	do not exercise	1 (7%)

### Baseline characteristics

In the ITT population, 7 of 15 subjects were male, the mean age was 58 ± 11 years, and the mean BMI was 24.9 ± 2.4 kg/m². All participants were Caucasian. Most of the subjects (12 participants) reported to have some chronic condition at the beginning of the study (7 participants with musculoskeletal and connective tissue disorder, 3 with cardiovascular disease, 2 with asthma, 4 with allergies, 1 with diverticulosis and 1 with overactive bladder disease). Clinical and biochemical parameters of the 15 study subjects at baseline and at end of study are shown in Table 4.

**Table 4:** **Clinical and biochemical parameters at baseline and end of study**

	**Baseline (n=15)**		**End of Study (n=15)**		
	**Mean ± SD**		**Mean ± SD**		***P***
Age (years)	57.9 ± 11.1				
Gender (male/female)	(8/7)				
Body weight (kg)	72.0 ± 10.9		71.7 ± 10.9		0.12
BMI	24.9 ± 2.4		24.8 ± 2.4		0.11
leukocytes (10^9^/l)	5.93 ± 1.63		5.63 ± 1.62		0.306
hemoglobin (g/l)	143.5 ± 9.9		140.2 ± 10.2		0.066
hematocrit (%)	43.9 ± 2.7		42.8 ± 3.0		0.066
erythrocytes (10^12^/l)	4.83 ± 0.37		4.66 ± 0.46		0.014
creatinine (μmol/l)	69.1 ± 10.9		69.7 ± 8.7		0.681
gamma-glutamyl transferase (U/l)	17.0 ± 5.6		14.8 ± 5.8		0.017

### Primary endpoint

The largest EPA + DHA incremental area under 72 h response curve in plasma phospholipids (iAUC_PL_) was detected after krill oil ingestion (mean 89.08 ± 33.36% × h). The iAUC_PL_ after krill oil ingestion was significantly larger than after ingestion of krill meal (mean 44.97 ± 18.07% × h, p < 0.001) or fish oil supplement (mean 59.15 ± 22.22% × h, p=0.003). Compared to krill meal, fish oil supplement showed no significantly larger iAUC_PL_ (p=0.036). Interestingly, if corrected for the dose given, the latter two iAUC_PL_ were identical.

**Figure 2:**
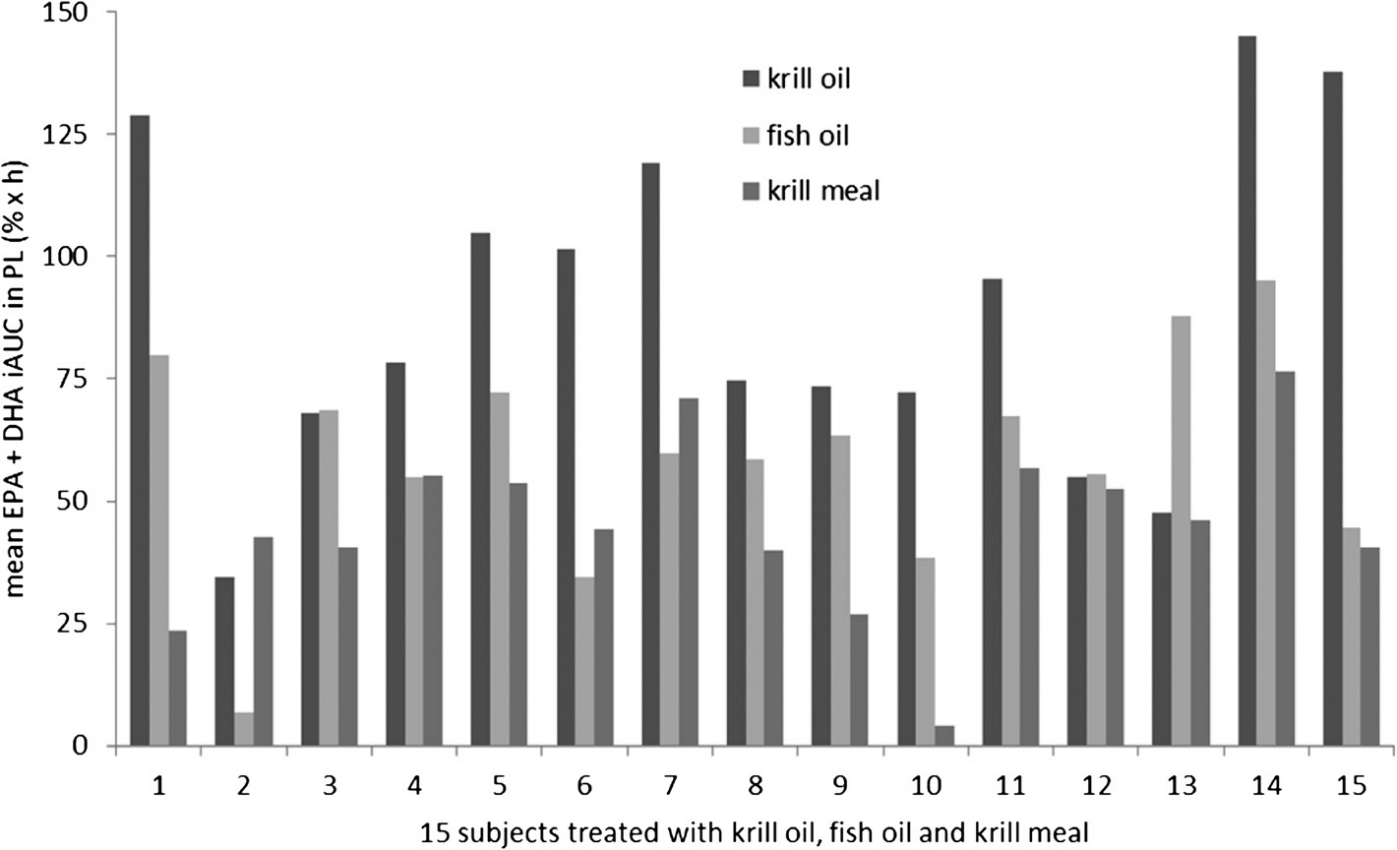
**Incremental area under 72 h response curve of EPA and DHA in plasma phospholipids after ingestion of different study products (krill oil, fish oil and krill meal) in 15 study subjects.**

As shown in Figure 2, there was a high variability in response for all study products in the 15 treated subjects. The minimum iAUC_PL_ detected after ingestion of krill oil was 3.45% × h, after fish oil 6.68% × h and after krill meal 4.09% × h. The maximum iAUC_PL_ detected after ingestion of krill oil was 144.92% × h, after fish oil 94.98% × h and after krill meal 76.57% × h. The maximum iAUC_PL_ levels after ingestion of the three different study products were all detected in subject number 14.

### Secondary endpoints

#### iAUC_TG_ in plasma triglycerides

**Figure 3:**
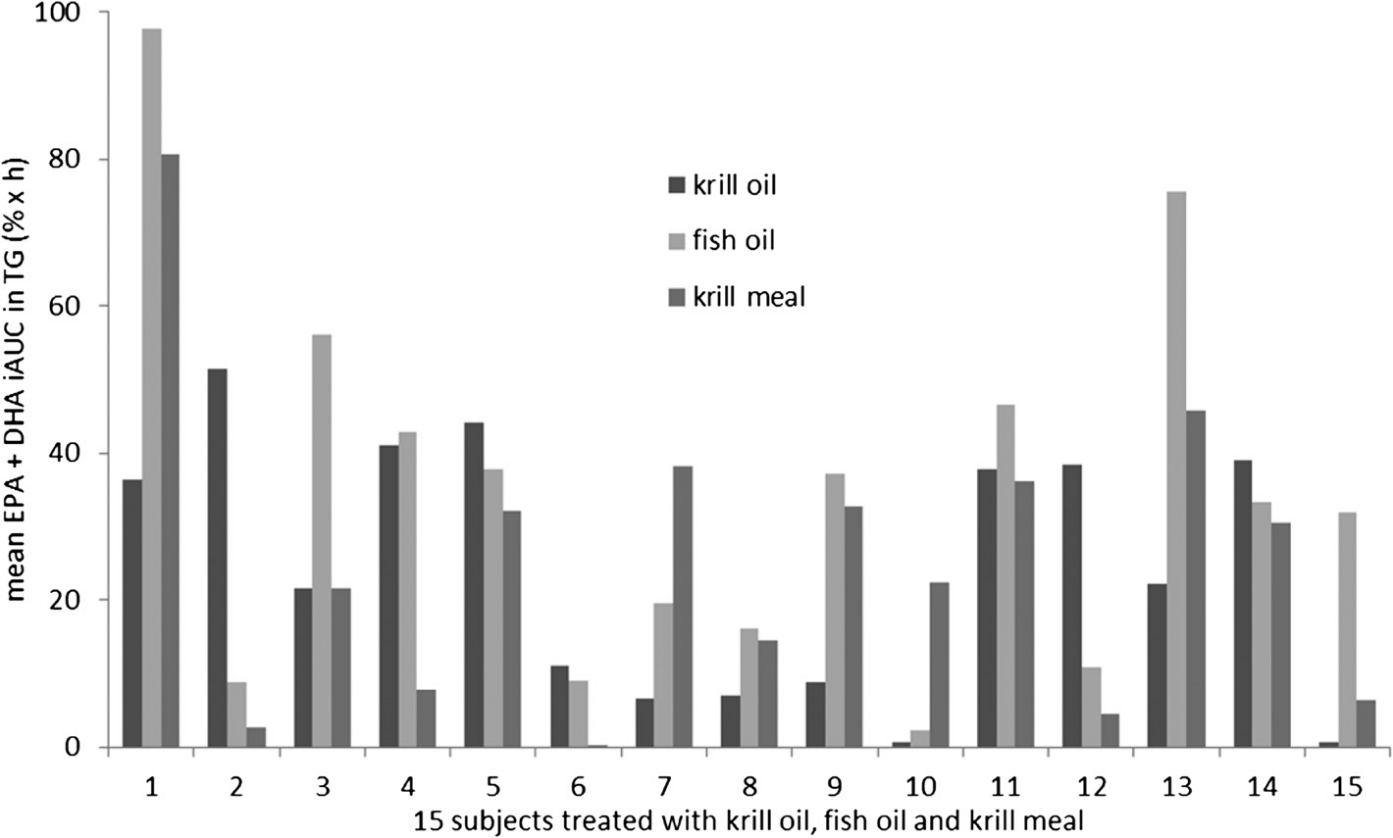
**Incremental area under 72 h response curve of EPA and DHA in plasma triglycerides after ingestion of different study products (krill oil, fish oil and krill meal) in 15 study subjects.**

Compared to the findings in plasma phospholipids, the mean EPA + DHA incremental area under 72 h response curve in plasma triglycerides (iAUC_TG_) showed lower values. Numerically, the largest iAUC_TG_ in plasma triglycerides was detected after fish oil supplement ingestion (mean 35.02 ± 26.54% × h), but the iAUC_TG_ after ingestion of krill oil (mean 24.46 ± 17.60% × h, p=0.165) or krill meal (mean 25.05 ± 21.18% × h, p=0.931) were not significantly smaller. As shown in Figure. 3, the EPA + DHA levels in plasma triglycerides demonstrated a high variability in response between the 15 study subjects. The minimum iAUC_TG_ detected after ingestion of krill oil was 0.56% × h, after fish oil 2.24% × h and after krill meal 0.05% × h. The maximum iAUC_TG_ detected after ingestion of krill oil was 51.49% × h, after fish oil 97.74% × h and after krill meal 80.63% × h.

#### EPA levels in plasma phospholipids

The baseline levels for the mean percentage of EPA in plasma phospholipids showed no significant difference for the 15 subjects before each intervention.

Numerically, the largest mean maximum increase of EPA compared to the baseline percentage of total fatty acids in plasma phospholipids (EPAc_max_) was detected after ingestion of krill oil (1.89 ± 0.38%), but this was not significantly higher than after fish oil (mean 1.07 ± 0.29%, p=0.044) or krill meal (mean 1.20 ± 0.20, p=0.628), which also held true for the comparison of fish oil and krill meal (p=0.017). The lowest EPAc_max_ for all three different study products were detected in subject number 10.

The time to maximum proportion of EPA in plasma phospholipids (EPAt_max_) showed significant differences between the three meals. After ingestion of krill meal, the mean EPAt_max_ was 13.33 ± 9.88 h. This was significantly shorter than compared to krill oil (mean 15.73 ± 15.60 h, p < 0.001) and to fish oil supplement (mean 24.53 ± 20.22, p=0.012). The EPAt_max_ after ingestion of krill oil was significantly shorter than after ingestion of fish oil supplement (p < 0.001).

#### DHA levels in plasma phospholipids

The baseline levels for the mean percentage of DHA in plasma phospholipids showed no significant difference for the 15 subjects before each intervention.

The maximum increase of DHA compared to the baseline percentage of total fatty acids in plasma phospholipids (DHAc_max_) was detected after ingestion of krill oil (mean 0.53 ± 0.22). This was significantly higher compared to krill meal (mean 0.26 ± 0.21, p=0.011) but not to fish oil supplement (mean 0.50 ± 0.27, p=0.769). After ingestion of fish oil supplement DHAc_max_ was significantly higher than after ingestion of krill meal (p=0.011). Compared to EPAc_max_, DHAc_max_ showed lower levels for each study meal (3.6 fold lower after krill oil, 2.1 fold lower after fish oil supplement and 4.6 fold lower after krill meal).

After ingestion of the three different study products the time to maximum proportion of DHA in plasma phospholipids (DHAt_max_) showed no significant difference for the three different study products. Compared to time to maximum proportion of EPA in plasma phospholipids (EPAt_max_), DHAt_max_ was 3.1 fold higher for krill oil, 2.2 fold higher for fish oil supplement and 2.9 fold higher for krill meal.

#### EPA + DHA levels in plasma phospholipids

Analysis of the EPA + DHA levels in plasma phospholipids at baseline showed no significant differences before intervention with the three study products.

The maximum increase of EPA + DHA compared to the baseline percentage of total fatty acids in plasma phospholipids (EPA + DHAc_max_) was detected after ingestion of krill oil (mean 1.89 ± 0.59%). This was significantly higher than after ingestion of fish oil supplement (mean 1.36 ± 0.47%, p=0.009) and after ingestion of krill meal (mean 1.12 ± 0.28%, p < 0.001). EPA + DHAc_max_ was not different between fish oil supplement and krill meal groups (p=0.083).

Time to maximum proportion of EPA + DHA in plasma phospholipids (EPA + DHAt_max_) showed no significant difference after ingestion of the three different study products.

#### EPA, DHA and EPA + DHA levels in plasma triglycerides

As shown in Table 5, EPA, DHA and EPA + DHA levels at baseline in plasma triglycerides showed no significant difference between the three study meals.

**Table 5:** **Results of fatty acid measurement in plasma phospholipids and triglycerides after intervention with different study products**

	**A krill oil**		**B fish oil**		**C krill meal**		**A:B**	**C:B**	**A:C**
	**(n=15)**		**(n=15)**		**(n=15)**				
	**Mean ± SD**		**Mean ± SD**		**Mean ± SD**		***p***	***p***	***p***
iAUC in PL (% x h)	89.08 ± 33.36		59.15 ± 22.22		44.97 ± 18.07		*0.003*	0.036	*<0.001*
iAUC in TG (% x h)	24.46 ± 17.60		35.02 ± 26.54		25.04 ± 21.18		0.165	0.035	0.931
EPA in PL at t0 (%)	1.29 ± 0.33		1.31 ± 0.36		1.32 ± 0.41		0.769	0.909	0.769
EPA cmax in PL (%)	1.89 ± 0.38		1.07 ± 0.29		1.20 ± 0.20		0.044	0.017	0.628
EPA tmax in PL (h)	15.73 ± 15.60		24.53 ± 20.22		13.33 ± 9.88		*<0.001*	*0.012*	*<0.001*
EPA max in PL (%)	3.17 ± 0.44		2.38 ± 0.42		2.52 ± 0.43		*<0.001*	0.108	*<0.001*
DHA in PL at t0 (%)	4.10 ± 0.81		3.98 ± 0.73		4.35 ± 0.90		0.249	0.023	0.172
DHA cmax in PL (%)	0.53 ± 0.22		0.50 ± 0.27		0.26 ± 0.21		0.796	*0.011*	*0.011*
DHA tmax in PL (h)	48.13 ± 23.72		54.53 ± 22.73		38.94 ± 26.12		0.041	0.796	0.090
DHA max in PL (%)	4.63 ± 0.73		4.48 ± 0.80		4.61 ± 0.84		0.090	0.230	0.866
EPA + DHA in PL at t0 (%)	5.39 ± 1.02		5.28 ± 0.91		5.67 ± 1.22		0.424	0.116	0.234
EPA + DHA cmax in PL (%)	1.89 ± 0.59		1.36 ± 0.47		1.12 ± 0.28		*0.009*	0.083	*<0.001*
EPA + DHA tmax in PL (h)	21.60 ± 17.92		19.09 ± 35.73		23.87 ± 19.07		0.021	0.044	0.718
EPA + DHA max in PL (%)	7.28 ± 0.88		6.65 ± 1.00		6.78 ± 1.16		*<0.001*	0.359	0.022
EPA in TG at t0 (%)	0.23 ± 0.12		0.22 ± 0.12		0.24 ± 0.17		0.851	0.650	0.728
EPA cmax in TG (%)	0.65 ± 0.30		0.64 ± 0.44		0.47 ± 0.24		0.919	0.081	0.030
EPA tmax in TG (h)	9.87 ± 17.26		11.20 ± 12.18		8.67 ± 6.44		0.820	0.372	0.742
EPA max in TG (%)	0.88 ± 0.32		0.87 ± 0.50		0.71 ± 0.33		0.876	0.068	0.039
DHA in TG at t0 (%)	0.80 ± 0.33		0.72 ± 0.53		0.75 ± 0.40		0.451	0.749	0.501
DHA cmax in TG (%)	0.54 ± 0.31		0.64 ± 0.52		0.50 ± 0.31		0.477	0.153	0.696
DHA tmax in TG (h)	18.00 ± 24.96		22.53 ± 21.59		18.40 ± 21.14		0.557	0.604	0.966
DHA max in TG (%)	1.34 ± 0.49		1.36 ± 0.75		1.25 ± 0.46		0.836	0.299	0.351
EPA + DHA in TG at t0 (%)	1.03 ± 0.42		0.95 ± 0.58		0.99 ± 0.50		0.446	0.679	0.674
EPA + DHA cmax in TG (%)	1.16 ± 0.55		1.18 ± 0.77		0.92 ± 0.44		0.882	0.104	0.151
EPA + DHA tmax in TG (h)	9.33 ± 17.45		19.73 ± 20.76		16.67 ± 19.65		0.020	0.637	0.281
EPA + DHA max in TG (%)	2.18 ± 0.74		2.13 ± 1.03		1.91 ± 0.73		0.777	0.127	0.092

After Bonferroni adjustment p values <0.017 were considered as statistically significant.

Also, the maximum increase of EPA, DHA and EPA + DHA compared to baseline percentage of total fatty acids, and the time to maximum proportion of EPA, DHA and EPA + DHA in plasma triglyceride showed no significant differences between the three intervention groups in plasma triglycerides.

A summary of fatty acid results in plasma phospholipids and triglycerides is shown in Table 5.

#### Adverse events

All adverse events reported by the participants were non-serious and non-severe. During the run-in period, only one subject had adverse events. At the intervention periods a total of nine adverse events were reported (1 after single administration of fish oil supplement, 5 after single administration of the krill meal and 3 after single administration of the fish oil supplement). Most of the adverse events were classified as mild. One subject reported decreased defecation frequency after ingestion of krill oil and krill meal and the symptom was classified as possibly related to the study product. Loose stools and heartburning were both reported by one participant after krill meal ingestion. Loose stools were classified as possible and heartburning as probable related to the study product. One subject reported prolonged bleeding time after blood sampling at the day when he ingested fish oil supplement. This symptom was classified as probably related to the study product.

#### Clinical and biochemical parameters

Clinical and biochemical parameters at beginning and at end of study are shown in Table 4. As shown in Table 4, the mean number of erythrocytes in blood decreased statistically significantly from 4.83 (0.37) to 4.66 (0.46) (p=0.014). The changes are considered to be related to the frequent blood sampling and not to the study products consumption. At the end of the study, the number of erythrocytes in blood was within reference range of the test in all participants and therefore the changes in the hematological tests are not considered as clinically significant.

The mean serum gamma-glutamyl transferase activity decreased from 17.0 (5.6) U/l to 14.8 (5.8) U/l (p=0.017), and might be due to chance. Serum creatinine concentration remained stable during the study.

## Discussion

This randomized open-label trial was conducted to compare the bioavailability of single doses of 1400 mg EPA plus DHA in a krill oil to 1102 mg EPA plus DHA in krill meal to 1455 mg EPA plus DHA in a fish oil in healthy volunteers in a 72 h timeframe. Profiles of EPA and DHA were measured in fatty acids of plasma phospholipids and triglycerides. We found a larger incremental area under the curve for EPA and DHA in plasma phospholipid fatty acids (primary endpoint) after krill oil, as compared to the other two sources of EPA and DHA.

By and large, plasma phospholipid fatty acid composition reflects dietary intake from hours to weeks, whereas triglyceride fatty acid composition reflects dietary intake from hours to days [5,9-12]. The evening before and during the first 24-hour period of each consumption of test products, participants consumed standardized meals. In addition, participants were instructed not to consume any other sources of EPA and DHA, and not to change the consumption of vegetable oil during the study. We conclude that acute changes observed in the measured fatty acids pools were attributable to the single administration of test products, rather than any other dietary changes.

The iAUC_PL_ after krill oil had a mean of 89.08 ± 33.36% × h, significantly larger than after ingestion of krill meal (mean 44.97 ± 18.07% × h, p < 0.001) or fish oil (mean 59.15 ± 22.22% × h, p=0.003). Our results are similar to a previous 4 week study, comparing identical doses of EPA plus DHA (600 mg) in krill oil vs. fish oil [13]. However, in one previous single dose study, differences failed to achieve significance [8]. Other studies of krill oil in humans did not compare identical doses, and are therefore difficult to compare [14,15]. One explanation for the better bioavailability of EPA and DHA in krill oil was that some krill oils contain substantial concentrations of EPA and DHA as free fatty acids, found in some, but not all, studies to have a better bioavailability than EPA and DHA in phospholipids [8,13-15]. The content of free fatty acids in krill oils varies, depending on a number of factors [16]. The content of free fatty acids in the krill oil used in this study, however, was low (2.6% of EPA and DHA as free fatty acids), supporting the view that the phospholipids, and not the free fatty acids, in krill oil are responsible for the higher bioavailability we found.

This view, however, is now challenged by our finding that the iAUC_PL_ after krill meal was non-significantly smaller than after fish oil, but was identical, if corrected for the dose given. While this finding was not reflected in the iAUC_TG_, it argues against the interpretation that the differences in bioavailability we found are due to differences in the chemical form of phospholipids vs. triglycerides, as the fat in krill oil and krill meal is identical. Rather, differences in the food matrix seem to be responsible. However, based on our data, we cannot provide a mechanistic explanation, why EPA + DHA had a better bioavailability in krill oil than in fish oil.

The response to study products varied largely from participant to participant (Figures 2 and 3). Before consumption of the study meals and capsules in the morning, participants were provided a standardized evening meal, followed by an overnight fast. This was done to minimize the large effects of concomitant food or fat intake observed in other studies [1,6]. Therefore, we suggest that the high interindividual variability in response in plasma phospholipids (iAUC_PL_ krill oil 3.45-144.92% × h; fish oil 6.68-94.98% × h; krill meal 4.09-76.57% × h) and plasma triglycerides (iAUC_TG_ krill oil was 0.56-51.49% × h, fish oil 2.24-97.74%, krill meal 0.05-80.63% × h) is not explained by the concomitant food intake or daytime of ingestion. All maximum iAUC_PL_-levels were detected in the same study subject, and in individuals 3, 12, and 13, krill oil did not display a higher bioavailability than fish oil. Taken together, our findings confirm the large inter-individual differences in uptake of EPA and DHA we and others observed earlier [5,17,18]. These differences remain to be explained mechanistically.

The variability of our findings on incorporation parameters into plasma phospholipid fatty acids, like maximum increase from baseline or time to maximum proportion of EPA, DHA or both combined, probably reflects the high inter-individual variability just discussed.

Overall, we found smaller AUC’s in response to all three forms of EPA + DHA in triglyceride fatty acids than in phospholipid fatty acids, and there was no significant difference among them. Generally, triglyceride fatty acids contained smaller percentages of EPA and DHA than phospholipid fatty acids [19]. The incorporation kinetics of our study support the interpretation that a smaller proportion of EPA and DHA is being incorporated into triglyceride fatty acids as one mechanism. Other mechanisms, like increased clearance, might also contribute. Clearly, however, incorporation into plasma phospholipid or triglyceride fatty acids does not appear to be a random phenomenon, but rather a regulated process.

Consistently, tmax of EPA in plasma phospholipids or plasma triglycerides occurred faster than tmax of DHA after all three preparations studied. However, both tmax of EPA and tmax of DHA had a shorter timeframe in plasma triglycerides than in plasma phospholipids. This argues against a simple crossing of large amounts of phospholipid or triglycerides molecule across the gastro-intestinal border, and for a differential handling of EPA and DHA during absorption among the preparations.

The single administration of krill oil and krill meal was well tolerated. Few subjects reported mild gastrointestinal symptoms after krill product ingestion. Clinical laboratory evaluation did not show any clinically significant changes in laboratory measurements.

### Strengths and limitations

A strength of our study is that the omega-3 preparations studied not only contained comparable doses of EPA and DHA in total but also of each fatty acid individually, which has not been done in all bioavailability studies of krill oil [4,5,7,8,13-15] Moreover, we used a cross-over design in order to limit the effects of the large inter-individual variability of fatty acid uptake [1]. Limitations of our study also exist: Although the capsules we used disintegrated in the stomach, we did not use identical numbers of identical capsules, as has been suggested recently for krill oil bioavailability studies [14]. However, although it cannot be excluded, it is difficult to envision a large impact of 16 vs 10 capsules or of 8 vs. 5 g fat ingested on the objective parameters we measured. Clearly, however, our results need to be substantiated in a longer-term trial using a longer-term parameter of omega-3 fatty acid bioavailability, like erythrocyte EPA and DHA, i.e. the Omega-3 Index [1].

## Conclusion

In a randomized, single-dose, single-blind, cross-over, active-reference trial in 15 healthy subjects, we compared the 72-hour bioavailability of approx. 1 700 mg EPA plus DHA in krill oil to krill meal and fish oil. Primary endpoint was the incremental area under the curve in plasma phospholipid fatty acids, reflecting recent dietary intake. According to the primary endpoint, EPA plus DHA had a higher bioavailability in krill oil, as compared to krill meal and fish oil. This was less well reflected in the secondary endpoints measured. Bioavailability of EPA plus DHA was not different between krill meal and fish oil, which argues against the interpretation that phospholipids are better absorbed than triglycerides. Our findings need to be substantiated in a longer-term trial using a parameter reflecting tissue EPA plus DHA.

## Abbreviations

EPA: Eicosapentaenoic acid
DHA: Docosahexaenoic acid
AUC: Area under the curve
PL: Phospholipids
TG: Triglycerides

## References

[1] von Schacky C (2015). Omega-3 Fatty Acids in Cardiovascular Disease-an Uphill Battle. Prostagland Leukotr Ess Fatty Acids.

[2] Plourde M, Cunnane SC (2008). Extremely limited synthesis of long chain polyunsaturates in adults: implications for their dietary essentiality and use as supplements. Appl Physiol Nutr Metab.

[3] Lane K, Derbyshire E, Li W, Brennan C (2014). Bioavailability and potential uses of vegetarian sources of omega-3 fatty acids: a review of the literature. Crit Rev Food Sci Nutr.

[4] Ulven SM, Kirkhus B, Lamglait A, Basu S, Elind E, Haider T (2011). Metabolic effects of krill oil are essentially similar to those of fish oil but at lower dose of EPA and DHA, in healthy volunteers. Lipids.

[5] Schuchardt JP, Hahn A (2013). Bioavailability of long-chain omega-3 fatty acids. Prostaglandins Leukot Essent Fatty Acids.

[6] Davidson MH, Johnson J, Rooney MW, Kyle ML, Kling DF (2012). A novel omega-3 free fatty acid formulation has dramatically improved bioavailability during a low-fat diet compared with omega-3-acid ethyl esters: The ECLIPSE (Epanova® compared to Lovaza® in a pharmacokinetic single-dose evaluation) study. J Clin Lipidol.

[7] Maki KC, Reeves MS, Farmer M, Griinari M, Berge K, Vik H (2009). Krill oil supplementation increases plasma concentrations of eicosapentaenoic and docosahexaenoic acids in overweight and obese men and women. Nutr Res.

[8] Schuchardt JP, Schenider I, Mayer H, Neubronner J, Von Schacky C, Hahn A (2011). Incorporation of EPA and DHA into plasma phospholipids in response to different omega-3 fatty acid formulations–a comparative bioavailability study of fish oil vs. krill oil. Lipids Health Dis.

[9] Harris WS, Varvel SA, Pottala JV, Warnick GR, McConnell JP (2013). The Comparative Effects Of An Acute Dose Of Fish Oil On Omega-3 Fatty Acid Levels in Red Blood Cells Versus Plasma: Implications for Clinical Utility. J Clin Lipidol.

[10] von Schacky C, Weber PC (1985). Metabolism and Effects on platelet function of the purified eicosapentaenoic and docosahexaenoic acids in humans. J Clin Invest.

[11] Sarkkinen ES, Ågren JJ, Ahola I, Ovaskainen M-L, Uusitupa MIJ (1994). Fatty acid composition of serum cholesterol esters, and erythrocyte and platelet membranes as indicator of adherence to fat-modified diets. Am J Clin Nutr.

[12] Matthan NR, Ip B, Resteghini N, Ausma LM, Lichtenstein AH (2010). Long-term fatty acid stability in human serum cholesteryl-ester, triglyceride, and phospholipid fractions. J Lipid Res.

[13] Ramprasath VR, Eyal I, Zchut S, Jones PJ (2013). Enhanced increase of omega-3 index in healthy individuals with response to 4-week n-3 fatty acid supplementation from krill oil versus fish oil. Lipids Health Dis.

[14] Salem N, Kuratko CN (2014). A reexamination of krill oil bioavailability studies. Lipids Health Dis.

[15] Laidlaw M, Cockerline CA, Rowe WJ (2014). A randomized clinical trial to determine the efficacy of manufacturers’ recommended doses of omega-3 fatty acids from different sources in facilitating cardiovascular disease risk reduction. Lipids Health Dis.

[16] Araujo P, Zhu H, Breivik JF, Hjelle JI, Zeng Y (2014). Determination and structural elucidation of triacylglycerols in krill oil by chromatographic techniques. Lipids.

[17] Köhler A, Bittner D, Löw A, von Schacky C (2010). Effects of a convenience drink fortified with n-3 fatty acids on the n-3 index. Br J Nutr..

[18] Muhlhausler BS, Gibson RA, Yelland LN, Makrides M (2014). Heterogeneity in cord blood DHA concentration: towards an explanation. Prostaglandins Leukot Essent Fatty Acids..

[19] Hodson L, Skeaff CM, Fielding BA (2008). Fatty acid composition of adipose tissue and blood in humans and its use as a biomarker of dietary intake. Prog Lipid Res..

